# Spatiotemporal Differentiation of the Coupling and Coordination of Production-Living-Ecology Functions in Hubei Province Based on the Global Entropy Value Method

**DOI:** 10.3390/ijerph192316062

**Published:** 2022-11-30

**Authors:** Yujie Liu, Jie Xu, Yong Zhou, Amat Muhtar, Li Wang

**Affiliations:** 1 Key Laboratory for Geographical Process Analysis & Simulation of Hubei Province, Central China Normal University, Wuhan 430079, China; 2 The College of Urban & Environmental Sciences, Central China Normal University, Wuhan 430079, China; 3 Faculty of Resources and Environmental Science, Hubei University, Wuhan 430062, China; 4 College of Life and Geography Sciences, Kashi University, Kashi 844006, China

**Keywords:** production-living-ecology functions, global entropy method, coupled coordination, territorial space, triangle model

## Abstract

Rapid urbanization and industrialization have brought about regional prosperity and pressure on the ecological environment, and the disorder of development has led to competition among the production-living-ecology functions. How to build livable cities, optimize the spatial layout of land, and promote the coordinated development of the production-living-ecology functions in various regions has become an important issue in the sustainable development and utilization of land space. In order to study the spatiotemporal conflict and coordination of the production-living-ecology functions with respect to the dramatic developments associated with the 21st century, this study took Hubei Province, which is the top-ranking economic region in China in recent years, as the study area and adopted the global entropy value method, triangle model, and coupled evaluation model to delineate an index system to measure the degree of conflict and coordination of the production-living-ecology functions in Hubei Province from 2000 to 2020, and also delineate zoning management based on statistical yearbook data. The results showed the following: (1) With respect to the time scale, the indices of the production-living-ecology functions in Hubei Province increased year by year, and the degree of coordination also increased yearly, from the stage of disorder with certain conflict to the stage of coordination with a high level of coupling. (2) On the spatial scale, the development of production-living-ecology functions in Hubei Province was unbalanced, which may be related to the overall development strategy of “two circles and one belt” in Hubei Province, with the eastern part of the province having a higher degree of coordination of the production-living-ecology functions and the western part having a lower degree of coordination. (3) Among the production-living-ecology functions, the ecological function of Hubei Province as a whole exhibited minimal change and maintained stable development, while the living and production functions underwent considerable development, indicating that Hubei Province has protected the orderly development of the ecological environment in the process of urbanization and new industrialization. (4) According to the development and coordination of the production-living-ecology functions in each region of Hubei Province, four development management zones were created: high-quality development zone, secondary development zone, balanced development zone, and development potential zone. Finally, policy suggestions are given for each zone.

## 1. Introduction

China is a vast country with uneven regional development; as a result, its resources are not evenly distributed over the national space [1]. With the rapid socioeconomic development, urbanization, and industrialization in China in recent years, the long-term economic- and production-oriented territorial space developed in the past has triggered conflicts and confusion among production, living, and ecological spaces [2]. The conflict among production, living, and ecological spaces has become increasingly intense, and the conflict among the production, living, and ecological functions, as the essence of the production-living-ecology spaces, has become more prominent [3]. The conflicts and incompatibilities among these three spatial functions have led to many problems, such as disorderly spatial development, ecological and environmental damage, resource depletion, and functional imbalance [4]. Therefore, it is important to carry out research on the functions of the production-living-ecology spaces. The study of production-living-ecology functions is an inevitable requirement for coordinated socioeconomic development in an ecological civilization, which necessarily guarantees the coordinated development of the region, and an important theoretical basis for the optimization of the spatial development pattern of national territories [5].

The living function, production function, and ecological function, referred to as the production-living-ecology functions, are the basic manifestations of the spatial functions of a region’s land and the products and services provided by the land under the combined influence of natural conditions and human activities. The ecological function provides ecological products and services for other functions, maintains ecological security and stability, and is the material source and environmental basis for the realization of the living and production functions [6,7]. The production function provides important products for human activities, such as industrial and agricultural products and transportation services [8,9,10], while the life function is the ultimate goal, providing human homes, recreational services, medical and educational services, and other direct necessities representing the quality of human life [11,12]. The functions of production, life, and ecology are mutually influential and interrelated, and do not exist as separate or unchanging entities [13,14]. Because of the complex competition and cooperation among the production-living-ecology functions, the Fifth Plenary Session of the 19th Central Committee clearly put forward a requirement to optimize the spatial pattern of land and promote the coordinated and sustainable development of the production-living-ecology functions [15]. The importance of these production-living-ecology functions is becoming increasingly prominent.

Both domestic and foreign scholars have conducted multi-faceted and systematic research related to production-living-ecology functions. Academics outside of China do not have a clear definition of the concept of these functions, and the relevant research has mainly focused on the connotation, definition, and derivation of multifunctional land [16,17]. The main research directions have included the definition and derivation of multifunctional land, the classification of multifunctional land systems, the definition and classification of multifunctional land index systems, and the definition and classification of multifunctional land function index systems [18,19]. Several studies have focused on the definition and derivation of multifunctional land; the classification of multifunctional land systems, focusing on the indicators and categories of multifunctional land; and the dynamics of subdivided multifunctional land use [20,21,22,23,24,25,26,27]. This study focuses on the evolution and optimization of multifunctional land use and the direction and evaluation of multifunctional land development. Domestic research on production-living-ecology functions has mainly been based on the study of land space, while some research has also focused on the study of multifunctional land, carried out in two directions: the study of the connotation and derivation of the production-living-ecology functions and the study of the dynamics of spatial and temporal scales. In the direction of the connotation and derivation of production-living-ecology functions, studies have mainly focused on the definition of the concept of production-living-ecology functions [28], including the theoretical framework of the evolution and characteristics of this concept [29,30] and the current zoning of utilization [31,32]. In the direction of research on the connotation and derivation of the production-living-ecology functions, studies have mainly focused on the definition of the concept of the production-living-ecology functions, including the theoretical framework of the evolution of this concept and the characteristics of the production-living-ecology functions, and the zoning of the current situation, including the spatial delineation of the production-living-ecology functions [33,34]. With respect to spatial and temporal scales, studies have mainly focused on the differentiation of the production-living-ecology functions on different spatial scales [35,36,37,38,39], such as urban clusters, administrative provinces, counties, or watersheds, and the characteristics of spatial and temporal evolution, specifically the evolution of the production-living-ecology functions [40,41,42]. The characteristics of spatiotemporal evolution include the spatiotemporal differentiation characteristics of the evolution of production-living-ecology functions, the coordinated evolution mechanism, and its influencing factors [43,44], specifically the influence of the sub-functions of the production-living-ecology functions on the production-living-ecology functions [45,46,47].

Current research on the production-living-ecology functions has made considerable achievements; however, there are still some shortcomings, including the fact that the majority of research has depended on the national space and not on provincial administrative regions. Moreover, few studies have explored the interrelationship among the production-living-ecology functions— most focus on the assessment of individual functions, and no in-depth research has been conducted on the interaction among the production-living-ecology functions. Hubei province, as the strongest province in central China, has become one of the fastest growing provinces in recent years due to its own geographical and humanistic advantages and policy factors, such as the shift of China’s center of development from the coast to the interior. The study of the differentiation and evolutionary characteristics of production-living-ecology functions in recent years is of great significance and has positive implications for territorial spatial planning, rural revitalization, ecological civilization construction, etc. It is also of great importance for the functional optimization of Hubei administrative regions, coordinated regional development, and policy orientation. This study took Hubei province as subject, and through analysis of the spatial and temporal conflicts and the degree of coordination among the production-living-ecology functions in this region during the period from 2000 to 2020, the relationship among the production-living-ecology functions was explored and the zoning was delineated according to the current situation in Hubei province. The results of this study will provide a reference and basis for the coordinated development of production-living-ecology functions in Hubei province.

## 2. Research Methodology and Overview

### 2.1. Global Entropy Value Method

Entropy, a term originally coined by the German physicist Clausius in 1850, is used to denote the degree of uniformity or disorder of an energy distribution in space; it is a physical concept of thermodynamics and a measure of the disorder (or order) of a system [48]. Later, Shannon proposed the concept of information entropy to quantify information. In system theory, the entropy method is the information management of “entropy”. The greater the entropy, the more chaotic the system, the greater the uncertainty, and the smaller the amount of information; conversely, the smaller the entropy, the more orderly the system, the smaller the uncertainty, and the greater the amount of information [49]. This method determines the weight of indicators based on the information provided by the observations of each indicator and avoids the interference of human factors, unlike the subjective assignment method [50,51,52,53]. Thus, the global entropy value method is an objective evaluation method that can integrate multiple indicators from multiple regions and multiple years [54,55]. The steps are as follows:

(i) Construct a global evaluation matrix. For N regions, T years, M indicators in the year by time to construct an NT×M global evaluation matrix X. The matrix consists of cross-sectional data tables for each year $X^{T}={\left(x_{nm}^{t}\right)}_{XT\times M}$:

(1) $$X={\left(X^{1},X^{2},X^{3}\ldots ,X^{T}\right)}_{XT\times M}={\left(x_{nm}^{t}\right)}_{XT\times M}$$

where $x_{nm}^{t}$ represents t years the region n in the evaluation matrix of m. The values of each indicator have the following ranges: 1<t≤T; 1<n≤N; and 1<m≤M.

(ii) Standardize the indicators. Different indicators will have a positive or negative influence on the system: the larger the value in positive indicators, the better; and the smaller the value in negative indicators, the better. Due to the differences between different indicators, they must undergo dimensionless standardization as follows:

Positive indicators:

(2) $$Z_{nm}=\frac{x_{nm}-minx_{m}}{maxx_{m}-minx_{m}}$$

Negative indicators:

(3) $$Z_{nm}=\frac{maxx_{m}-x_{nm}}{maxx_{m}-minx_{m}}$$

where $Z_{nm}$ is the $x_{nm}^{t}$ of the standardized index values of $minx_{m}$ and $maxx_{m}$, which are the minimum and maximum values of m, the index value in the region.

(iii) Calculate the percentage of indicators, the first n region in m. The share of the value of the indicator in the matrix $y_{nm}$ is:

(4) $$y_{nm}=\frac{Z_{nm}}{\sum_{n=1}^{NT}Z_{nm}}$$

(iv) Calculate the information entropy value of the first m. The information entropy of the value of the index $e_{m}$ is:

(5) $$e_{m}=-\frac{1}{\ln NT}\sum_{n=1}^{NT}y_{nm}\ln y_{nm}$$

(v) Calculate the information utility value of the first m. The information utility value of the first indicator value $d_{m}$ is:

(6) $$d_{m}=1-e_{m}$$

(vi) Calculate the indicator weights. The weight of the first indicator value $W_{m}$ is:

(7) $$W_{m}=\frac{d_{m}}{\sum_{n=1}^{M}d_{m}}$$

(vii) Calculate the comprehensive evaluation value of the first n. The comprehensive evaluation value of the first area $U_{n}$ is:

(8) $$U_{n}=\sum_{n=1}^{M}y_{nm}W_{m}$$

### 2.2. The Construction of the Production-Living-Ecology Function Index System

In this study, the relationships among the production-living-ecology functions were examined, and three first-level indicators—the production function index (PFI), living function index (LFI), and ecological function index (EFI)—were used to represent the development of production, living, and ecological functions, respectively. The index system was determined due to the relevance and difficulty of data acquisition, referencing the papers of De Zhou et al. [56,57], based on the data requirements of the global entropy value method, and the combination of ideas, such as resource-carrying capacity, to study the relative levels among the production-living-ecology functions. In order to increase the number of index columns as much as possible so as to obtain more information, therefore, the production function index, life function index, and ecological function index are selected as much as possible, with 6, 12, and 6 three-level indicators, respectively. Furthermore, this paper mainly studies the changes of the relative levels among the production-living-ecology functions, and the size of the relative weights has little influence on the research weights and research results. Although the number of indicators varies in the index system, the decisive role in the entropy value method is the degree of fluctuation of the single column indicators, and in addition, the size of the single column and the sum index weights can also explain the interaction levels of the production-living-ecology functions to some extent.

The production function index consists of three secondary indicators, including the agricultural production function (F1), which represents the level of agricultural development; the economic growth function (F2), which represents the level of economic development; and the traffic function (F3), which represents the traffic-carrying capacity. The life function index consists of the employment security function (F4), representing the social working population and income; the social security function (F5), representing social input and guarantee per capita; the habitat function (F6), representing the input and construction level of the living environment; the entertainment function (F7), representing the spiritual, material, and cultural supply of the people; the science and education function (F8), representing social science and education input; and the medical security function (F9), representing the medical care level of the region. The ecological function index is composed of two secondary indicators: the resource security function (F10), which represents the human living environment and resource-carrying level, and the ecological balance function (F11), which represents the land output and environmental pollution level. Each secondary index is composed of corresponding tertiary indices.

**Figure 1:**
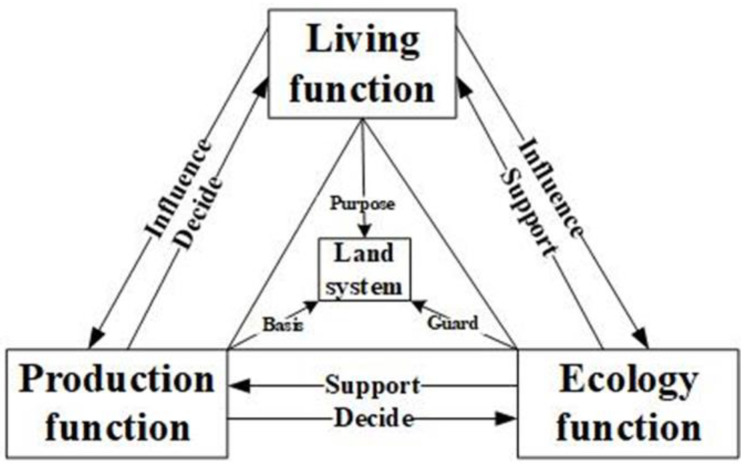
The interactions among the production-living-ecology functions.

The corresponding objective weights of all the indices and the production-living-ecology function indices were calculated according to the global entropy value method, as shown in Table 1; a diagram of the interrelationships among the production-living-ecology functions was also obtained, as shown in Figure 1.

### 2.3. Triangle Model

**Figure 2:**
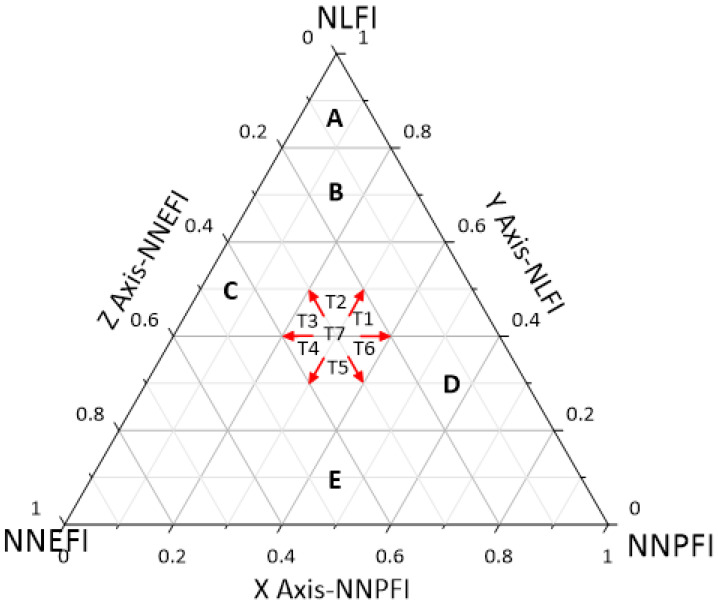
Triangle model diagram of the relative status and trend of the production-living-ecology functions. Abbreviations: LFI—normalized living function index; NPFI—normalized non-production function index; NEFI—normalized non-ecology function index; NEFI—normalized non-ecology function index.

The triangle model is a qualitative classification tool in soil science known as the international system of soil texture triangulation, which determines the types of soil by the ratio of the content of three particles: sand, powder, and clay (the values of the three add up to one). This model has recently been applied in several other fields, such as economics, geography, environmental science, etc. [58,59,60]. In this study, the complementary relationship among the three functional indices (LFI, PFI, and EFI) calculated above does not necessarily exist and cannot be represented by the triangle model [61]. The triangle model is a model that considers the balance degree of the development level of three types of indicators. The apex of the triangle is the theoretical maximum of the indicator. At the same time, due to the large differences in the value domains of the three functional indices in the results of the global entropy method, in order to construct complementary relationships among these indices, it is necessary to convert the data into a form that can be interpreted by the triangular model. Firstly, through linear normalization, the LFI, PFI, and EFI were normalized to the normalized life functional index (NLFI), normalized production functional index (NPFI), and normalized ecological functional index (NEFI), respectively, with value domains of 0–1. In addition, two new normalized indices, a non-production function index (NNPFI = 1 − NPFI) and normalized non-ecological function index (NNEFI = 1 − NEFI), as well as two new indicators for the normalized non-production function index (NLFI = 1 − NPFI) and the normalized non-ecological function index (NNEFI = 1 − NEFI), were introduced. These three indices match the top of the triangle in the triangle model. Thus, by drawing a complementary relationship among NLFI, NNPFI, and NNEFI, the relative states and trends of the production-living-ecology functions were represented by a triangle model (using the Grapher software, which sums the three to one), as in Figure 2. In the figure, the production-living-ecology functions are shown in an equilateral triangle, with NLFI at the top, NNEFI at the bottom left, and NNPFI at the bottom right. In addition, the Z, Y, and X axes are oriented in counterclockwise direction from 0 to 1 to divide the relative values among the three based on Table 2, which is divided into five regions, representing the five levels of the relative status quo of the tristimulus function, and Table 3, which is divided into the seven directions of the evolutionary trend of the tristimulus function.

### 2.4. Coupled Evaluation Model

The coupled evaluation model is used to test the level of coordinated development between multivariate systems and includes two indicators: the coupling degree and coupling coordination degree [62,63,64,65]. In this study, the coupling degree refers to the degree of coordinated development of the mutual influence among the production-living-ecology functions, while the coordination degree refers to the degree of benign coupling in the process of mutual influence among the production-living-ecology functions—its magnitude reflects the level of coordinated development among the production-living-ecology functions. The coupling degree is calculated by the following formula:

(9) $$C_{i}=3\frac{\sqrt[{3}]{LFI_{i}\times PFI_{i}\times EFI_{i}}}{LFI_{i}+PFI_{i}+EFI_{i}}$$

where i is the number of samples; $LFI_{i}$, $PFI_{i}$, and $EFI_{i}$ are the values of the i production function index, life function index, and ecological function index values, respectively, of the first region (if $LFI_{i}$, $PFI_{i}$, and $EFI_{i}$ are too different from each other; the index values should be dimensionless to ensure that they are in the range of (0, 1)); and $C_{i}$ is the coupling degree among the production-living-ecology functions, i.e., i. The value range of the coupling degree is (0, 1): the closer the value is to 0, the lower the coupling degree among the production-living-ecology functions, while the closer the value is to 1, the higher the coupling degree among the production-living-ecology functions. The values can be divided into four levels according to their size, as shown in Table 4.

The coupling coordination degree is calculated as follows:

(10) $$T=\alpha LFI_{i}+\beta PFI_{i}{}_{i}+\gamma EFI_{i}$$

(11) $$D_{i}=\sqrt{C_{i}\times T}$$

where T is the coordination index; α, β, and γ are the weights of the tristimulus functional index in the system as a whole, where α+β+γ = 1; and $D_{i}$ is the coupling coordination degree, taking values in the range of (0, 1), representing the degree of benign coupling development among the production-living-ecology functions in the system. The coupling coordination degrees can be divided into 10 levels according to their values, as shown in Table 5.

## 3. Study Area and Data Sources

### 3.1. Overview of the Study Area

**Figure 3:**
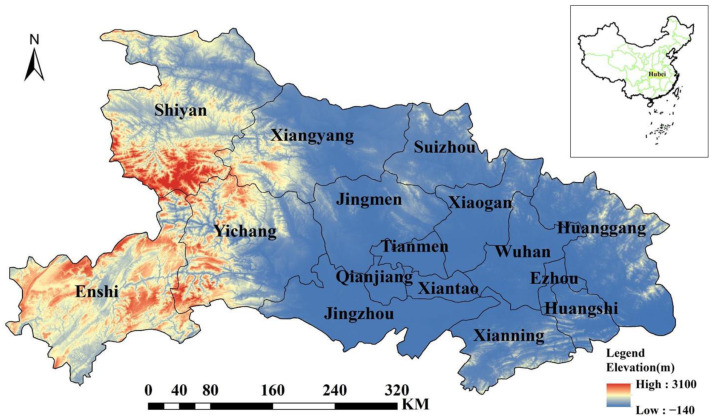
Geographic location and elevation distribution of Hubei Province.

Wuhan, the capital of Hubei Province, is located in the middle reaches of the Yangtze River in the central hinterland of China. It is north of Dongting Lake and adjacent to six provincial administrative regions—Anhui Province, Chongqing Municipality, Shaanxi Province, Jiangxi Province, Hunan Province, and Henan Province—between 29°01′53″–33°6′47″ N and 108°21′42″ E. Its total area is about 186,000 square kilometers, accounting for about 2% of China’s total land area. Hubei Province is surrounded by mountains from the east to west and north to west, and the central part is a low and incomplete basin with many rivers and lakes, whose geographical location and elevation distribution are shown in Figure 3. By 2021, Hubei Province will have a resident population of 58.3 million and achieve a regional GDP of CNY 500.1294 billion. It should be noted that Shennongjia Forest District is a county-level administrative region originally belonging to Shiyan City. Due to its close economic, political, and cultural relationship with Shiyan City and its special nature as a forest district, it does not have the function of a complete municipal administrative region, has few data, and has a sparse population; thus, it was incorporated into the administrative region of Shiyan City for this analysis.

### 3.2. Data Source and Pre-Processing

The main socioeconomic data used in this study were obtained from the Hubei Provincial Statistical Yearbook 2000–2020, Hubei National Economic and Social Development Statistical Bulletin 2000–2020, Hubei Rural Statistical Yearbook 2000–2020, Hubei Urban Statistical Yearbook 2000–2020, and Hubei Ecological Environment Bulletin 2000–2020. Carbon emission data were obtained from a study by Xiao Pengnan et al. [66], and elevation data were obtained from the Geospatial Data Cloud (URL: http://www.gscloud.cn/; accessed on 1 January 2020). In this study, EvaGear (Version 2.4) was used for processing the global entropy method, the triangle model was calculated in Grapher (Version 16), and the figure processing was performed in ArcMap (Version 10.2) and Visio (Version 2019).

## 4. Results and Analysis

### 4.1. Characteristics of Changes in the Production-Living-Ecology Functions of Hubei Province

Based on calculations using the global entropy value method, an index system was constructed and the three functional indicators and corresponding weights of Hubei Province were obtained, as shown in Table 1, among which the negative indicators were population density, sulfur dioxide emissions per capita, fertilizer application per hectare, and carbon emissions per capita. As can be seen from Table 1, the weights of the primary indicators were 0.6403, 0.3229, and 0.0369 for LFI, PFI, and EFI, respectively. In this study, the LFI indicators had relatively large weights, while the EFI indicators had relatively small weights, because according to the objective assignment of the global entropy value method, the life-related indicators in Hubei Province from 2000 to 2020 exhibited great variation and rapid development. The production-related indicators showed large variation and fast development, while the ecological-related indicators exhibited very little variation. Compared to the highly variable life- and production-related indicators, the ecological indicators remained in a relatively stable state. Therefore, according to the objective of information entropy, among the production-living-ecology functions in Hubei Province from 2000 to 2020, the ecological function itself showed minimal variation and maintained stable development, while the life and production functions underwent considerable development.

**Figure 4:**
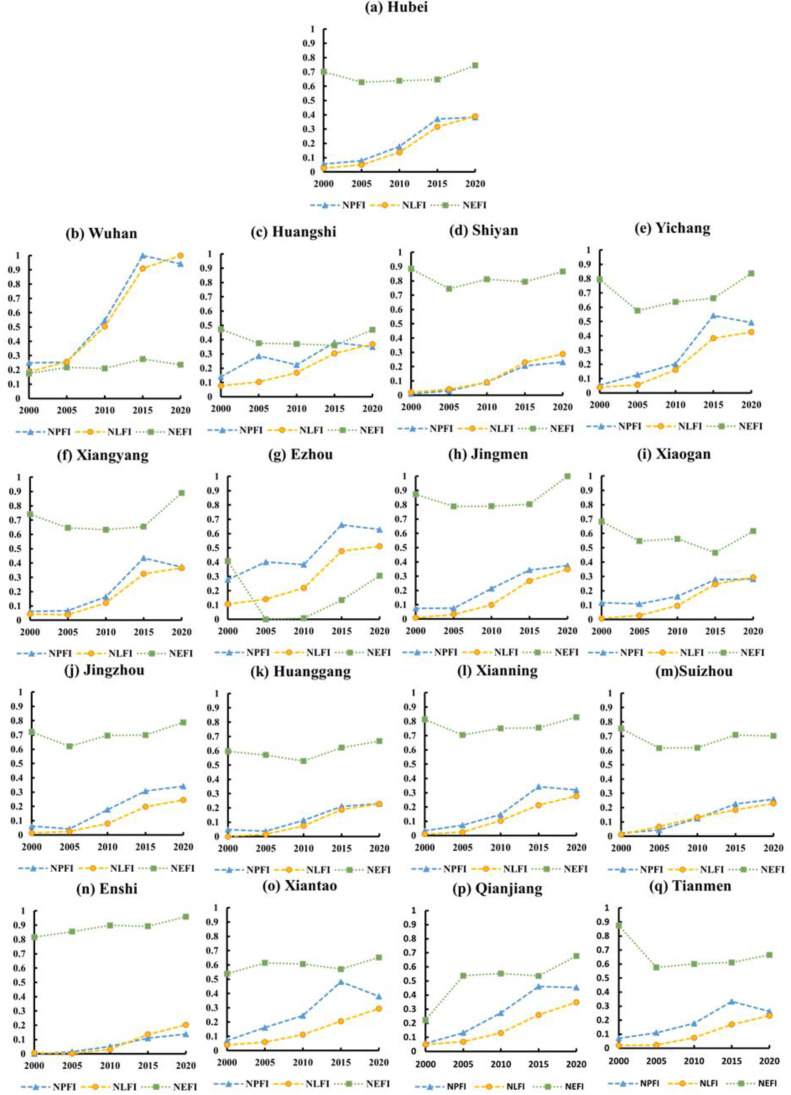
Changes in the production-living-ecology functions in Hubei Province from 2000 to 2020.

Because the LFI, PFI, and EFI values obtained by the global entropy weighting method varied greatly, in order to visualize the relative changes in the characteristics of the production-living-ecology functions in Hubei Province from 2000 to 2020, these values were linearly normalized to obtain NLFI, NPFI, and NEFI values (see Figure 4). From Figure 4, it can be seen that the ecological function of Hubei Province decreased slightly from 2000 to 2005; from 2015 to 2020, the ecological function of Hubei Province increased more significantly; and from 2005 to 2010, the ecological function of Hubei Province remained basically the same, with no significant changes. The overall development of the living and production functions occurred simultaneously, and the rate of development from 2000 to 2015 became increasingly fast; in addition, the production function almost stagnated in the period of 2015–2020, while the living function continued to develop minimally. In China, the national regional development strategy is gradually tilting from the coast to the mainland. Hubei Province is located in the center of China’s map, with no more than two provinces between it and any other province, and it sits on the Yangtze River waterway and Han River waterway and has developed transportation. Hubei Province has seen a considerable development trend in recent years; thus, it is significant to study the direct conflict and coordination of its production-living-ecology functions. In 2000, Hubei Province was still in the process of implementing the “2000 Implementation Opinions of Large and Medium-sized State-owned Loss-making Enterprises” and emerging from an economic quagmire, during which the ecological function declined and the production and living functions increased slightly. In 2005, the Wuhan Railway Bureau was established and the development of the railroad network began, which led to economic and social development; during this period, the functions of living and production increased more rapidly. In 2009, Hubei Province made the decision to open the Hubei Yangtze River Economic Belt, which marked the formation of the overall strategy of Hubei as “two circles and one belt”. From then on, Hubei Province entered a period of rapid development, and the life and production functions were greatly enhanced. Hubei Province implemented the quality development program in 2015 and proposed the Yangtze River protection in 2017 to ensure the simultaneous protection of the ecological environment. At the end of 2019, due to the impact of the new coronavirus epidemic, the economy fell into a stagnant phase and development slowed down. During this period, the development of the ecological and production functions almost stagnated, while the life function developed minimally.

From Figure 4, looking at the general trend of the change in the production-living-ecology functions in Hubei Province, it can be seen that the development of the production-living-ecology functions in each city showed some differences. In Wuhan, Ezhou, and Huangshi, the ecological function was lower, probably because these three areas have the highest overall urbanization level and only one agricultural county in total; thus, the ecological function is in a weaker position. Wuhan and Ezhou had stronger living and production functions. Wuhan—as the provincial capital—is more powerful in siphoning from other cities in Hubei Province, is the main industrial center and economic center, and is also the most urbanized area. Ezhou, as a weaker city in Hubei Province, has a very small administrative area and shares a large border with Wuhan; its industry and economy are mainly influenced by the radiation of Wuhan, thus its living and production functions were higher. In addition, Yichang and Xiangyang had higher living and production functions, likely because Yichang is in the center of the Yangtze River Economic Belt, which—with the Gezhou Dam and Three Gorges Dam—has well-developed transportation and good conditions for socioeconomic development, and Xiangyang is in the northwestern part of Hubei Province, located in a sub-node of the railroad network, which connects the north and south, with a good industrial base and developed agriculture.

### 4.2. Characteristics of Changes in the Interaction of the Production-Living-Ecology Functions in Hubei Province

**Figure 5:**
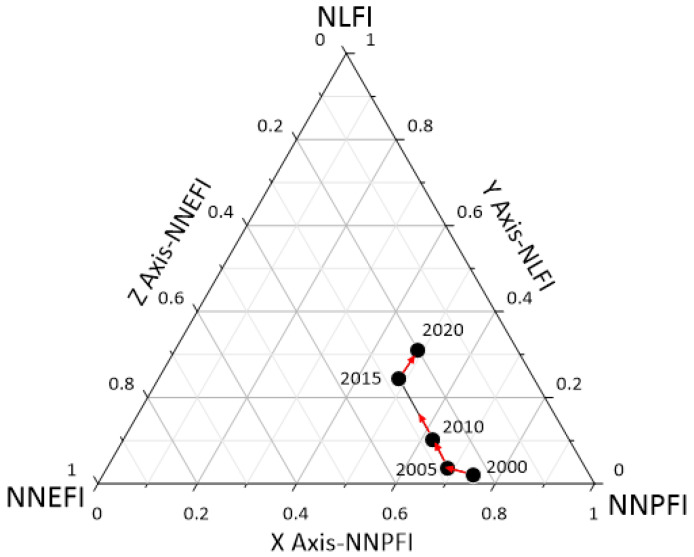
Interaction of the production-living-ecology functions in Hubei Province, 2000–2020.

From Figure 5, it can be seen that the development of the interactions among the production-living-ecology functions in Hubei Province took place in two stages, with a low level (Zone E) observed from 2000 to 2010 and a lower level (Zone D) recorded from 2015 to 2020, although the level has been increasing. In terms of the development trend, the interaction among the production-living-ecology functions in Hubei Province showed a general upward trend in 2000–2005 and 2015–2020 and a strong upward trend in 2005–2010. In general, the interaction among the production-living-ecology functions in Hubei Province showed an upward trend, and the upward trend was slow, then fast, and finally slow, and the production-living-ecology functions tended to be in balance with each other.

**Figure 6:**
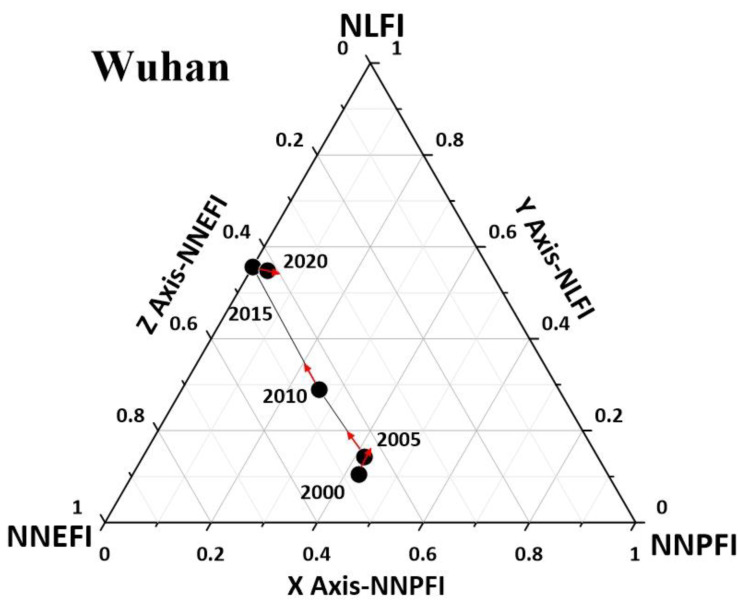
Map of interaction level C of the production-living-ecology functions at the municipal level in Hubei Province.

**Figure 7:**
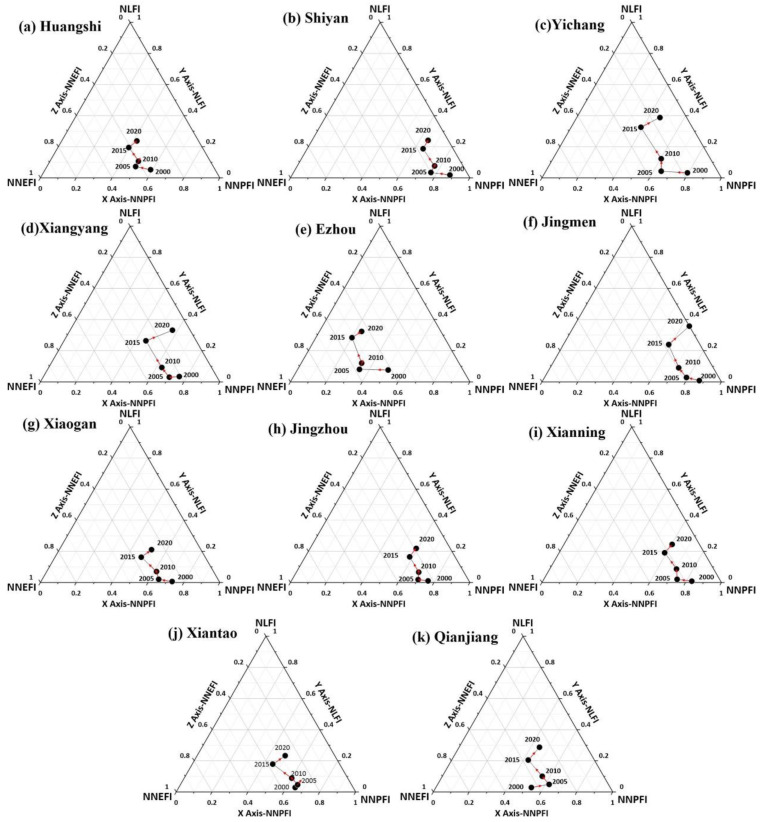
Map of the level of interaction among the production-living-ecology functions at the municipal level in Hubei Province (level D).

**Figure 8:**
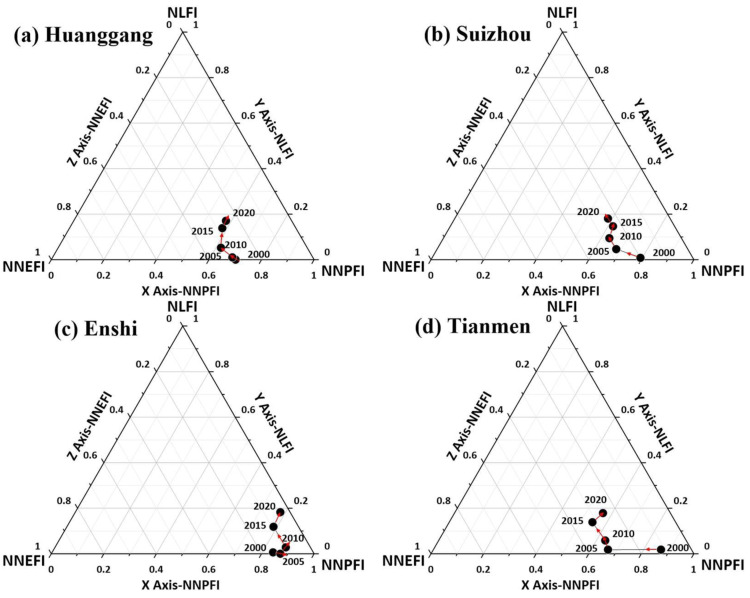
Map of the level of interaction among the production-living-ecology functions at the municipal level in Hubei Province (level E).

The characteristics of the changes in the interaction of the production-living-ecology functions in each municipal administrative district in Hubei Province were roughly the same as those in Hubei Province, where the developmental stages roughly showed development from a low level to a high level and development exhibited an upward trend that showed certain differences in time and space. Based on the final level of the interaction of the production-living-ecology functions in each municipal administrative district, they were divided into three categories—Zone C, Zone D, and Zone E—and Figure 6, Figure 7 and Figure 8 were obtained.

As can be seen from Figure 6, in terms of developmental stages, high-level interactions among Wuhan’s production-living-ecology functions were observed from 2015 to 2020 (Zone C), while the level was higher in 2000–2010 relative to other regions. Wuhan, as the provincial capital of Hubei Province, is economically and socially developed, and its living and production functions are relatively advanced at this stage, while its ecological functions are underdeveloped. In terms of developmental trends, from 2000–2015 Wuhan region has been in an upward trend of the production-living-ecology functions, including a strong upward trend from 2000–2005 and 2010–2015, and a general upward trend from 2005–2010. The interactions among Wuhan’s production-living-ecology functions showed a general downward trend in 2015–2020, mainly due to the impact of the new coronavirus epidemic. What mainly plagues Wuhan’s development is its lower level of ecological function.

As can be seen from Figure 7, the 2020 life function interactions in Huangshi, Shiyan, Yichang, Xiangyang, Ezhou, Jingmen, Xiaogan, Jingzhou, Xianning, Xiantao, and Qianjiang were at a low level (Zone D), indicating that the triple function interaction levels, and thus development, in these 11 regions were relatively average for Hubei Province. In addition, four regions, Yichang, Xiangyang, Ezhou, and Jingmen, exhibited relatively high interaction levels, indicating that these four regions had high development levels for the production-living-ecology functions, second only to Wuhan. Among them, the rising trends of these regions are slower from 2000–2005 and 2015–2020, and stronger from 2005–2015.

As can be seen from Figure 8, the interaction of the production-living-ecology functions in Huanggang, Enshi, Suizhou, and Tianmen was always at a low level (E area), especially in the lower right corner of the production-living-ecology functions in Enshi. This suggested that its life production functions were all at very low levels, and the levels in other regions were also low, indicating that the development level of the production-living-ecology functions in these four regions is very low. This is likely because three of these regions, including Huanggang, Enshi, and Suizhou, are located on the borders of Hubei Province and thus have a poor industrial base, inconvenient transportation, and hindered economic development. Tianmen is located in the center of Hubei Province but experiences a similar situation. Coupled with no policy leaning and no advantageous industrial support, this has led to the development center of the surrounding areas moving towards Jingmen, Xiantao, and other areas. However, the development trend of these areas is good, such as Huanggang, and Enshi from 2015–2020 demonstrated a strong upward trend, with considerable development potential.

### 4.3. Analysis of the Coupled Coordination Model of the Production-Living-Ecology Functions in Hubei Province

**Figure 10:**
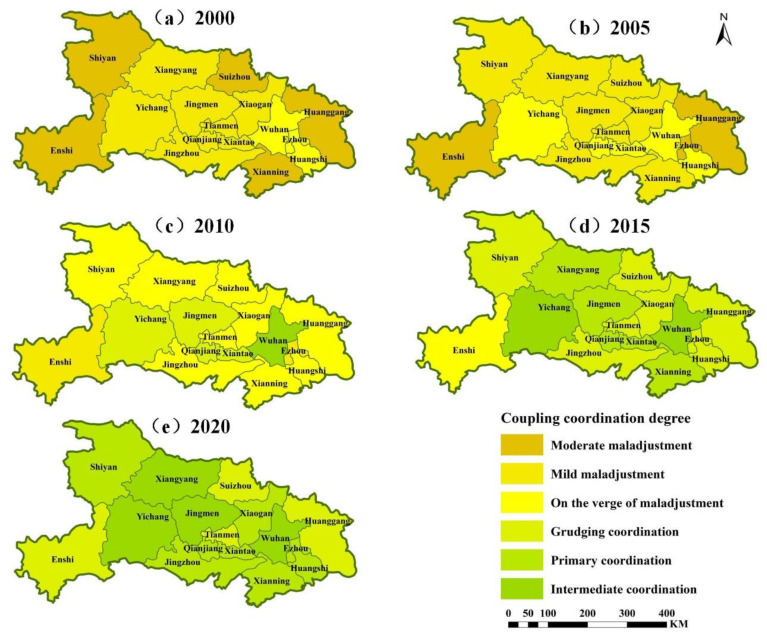
Change in the coupling coordination of production-living-ecology functions at the municipal level from 2000 to 2020 in Hubei Province.

By referring to the levels shown in Table 2 and Table 3 for the model analysis of the coupling and coordination of the production-living-ecology functions in Hubei Province from 2000 to 2020, Figure 9 and Figure 10 and Table 6 were obtained. The coupling degree reflects the magnitude of interaction and the degree of relationship among the production-living-ecology functions in Hubei Province. From the spatial perspective, the overall coupling degree in Hubei Province increased from 0.44 to 0.952, developing from the antagonistic stage to the high-level coupling stage. The coupling degree was relatively high in the eastern region, mainly Wuhan, and relatively low in the western region. From the perspective of time, in 2000, the spatial differences in the coupling degree of the production-living-ecology functions in Hubei Province were large, with an overall range of 0.184–0.989, exhibiting all four stages. In general, the antagonistic stage and the high-level coupling stage were regionally distributed in the eastern and central parts of Hubei Province, the abrasive stage was distributed in the east, the antagonistic stage was distributed throughout most of Hubei Province, and the low-level coupling stage was distributed in the western and northern parts of Hubei Province. Among them, Enshi had the lowest coupling degree and Wuhan had the highest coupling degree. In 2005, the spatial variation in the coupling of the production-living-ecology functions in Hubei Province was large with four stages, with the overall range of 0.207–0.997, but mainly the antagonistic stage and the grinding stage were distributed throughout most of Hubei. Meanwhile, the high-level coupling stage was only found in Wuhan and Huangshi in the east, and the low-level coupling stage was only found in Enshi, which was the lowest coupling degree in the southwest. In addition, Wuhan had the highest degree of coupling. In 2010, the spatial variation in the coupling of the production-living-ecology functions in Hubei Province was reduced with only three stages, with the overall range of 0.392–0.952, mainly the grinding stage, distributed throughout most of Hubei, while the high-level coupling stage was distributed in the eastern and central/western parts of Hubei Province and the antagonistic stage was only found in Enshi, which was the lowest coupling degree. In addition, Huangshi had the highest coupling degree. From 2015 to 2020, the coupling degree increased slightly, with the overall range from 0.643–0.996 to 0.704–0.992, but it generally remained unchanged. The whole of Hubei Province entered the high-level coupling stage, except for Enshi, which was in the grinding stage. Among them, the lowest coupling degree always was in Enshi, and the highest coupling degree always was in Huangshi.

The coupling coordination degree reflects the degree of benign coupling among the production-living-ecology functions in Hubei Province, that is, the degree of mutual promotion of development. From the spatial perspective, the overall coupling coordination degree of Hubei Province improved from 0.342 to 0.694, developing from mild disorder to primary coordination; the coupling coordination degree of the eastern region, mainly Wuhan, was relatively high, followed by the central region, while that of the western region was relatively low. From the perspective of time, the spatial difference in the coupling coordination of the production-living-ecology functions in Hubei Province in 2000 was small, with an overall range of 0.226–0.487 and with three degrees, from moderate disorder to near disorder, dominated by disorder. Near disorder dominated in eastern Hubei, mild disorder in central Hubei, and moderate disorder in western, northern, and eastern Hubei, where the area with the lowest coupling coordination was Enshi and the highest coupling coordination was Ezhou. In 2005, the coupling coordination of the production-living-ecology functions in Hubei Province was slightly improved compared to 2000. The overall range was 0.247–0.497, but still showed degrees from moderate disorder to near disorder (mainly disorder). However, the number of areas with moderate disorders decreased in Enshi in the southwest and Huanggang and Ezhou in the east, while the number of areas with near disorders increased in the east and central/western parts of Hubei. The area with the lowest degree of coordination was Enshi, and the area with the highest degree of coupling coordination was Wuhan. In 2010, the spatial differences in the coordination of the coupling of the production-living-ecology functions in Hubei increased compared to previous years, a coordination stage appeared, and the overall range was 0.341–0.625, with four stages in total from mild disorders to primary coordination. In 2010, the highest degree of coupling coordination of primary coordination was distributed in Wuhan in the east, and barely coordinated was distributed in the central and western part of Hubei Province, while the area with the lowest coupling coordination was Ezhou. In 2015, the overall coordination of the coupling of the production-living-ecology functions in Hubei Province increased, with an overall range of 0.495–0.793 and with four stages, from near disorder to intermediate coordination, mainly in the coordination stage. Intermediate coordination was distributed in Wuhan in the east and Yichang in the west, where the highest coupling coordination was Yichang, while near disorder was found only in Enshi, which had the lowest coupling coordination degree in the western part of Hubei Province. In 2020, the overall coordination of the coupling of the production-living-ecology functions in Hubei Province increased again, with an overall range of 0.553–0.779 and with three stages, from near disorder to intermediate coordination, mainly in the coordination stage. Intermediate coordination was distributed in the east, central, and western parts of Hubei Province, while primary coordination was distributed throughout most of Hubei Province, where the region with the lowest coupling coordination was Enshi, and the region with the highest coupling coordination was Wuhan.

In terms of the comprehensive coupling degree and coupling coordination, the degree of coordination of the production-living-ecology functions in Hubei Province increased at an average rate from 2000 to 2005, increased at a rapid rate from 2005 to 2015, and slowed down from 2015 to 2020. The degree of coupling and coordination of the production-living-ecology functions in Hubei province evolved toward a high level, and there were significant differences in the degree of coupling and coupling coordination in different regions, among which the higher regions were Wuhan, Huangshi and Yichang, and the lower regions were Enshi and Shiyan.

### 4.4. Development Management Zoning

**Figure 11:**
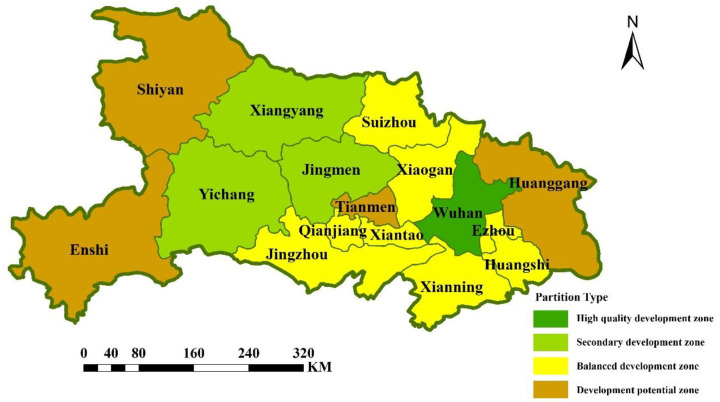
Hubei Province zoning management map.

Combining the global entropy value method, triangle model, coupling coordination model evaluation, and the actual situation in Hubei Province, the division levels were determined, as shown in Table 7, and Hubei Province was divided into four types of development management zones, namely, high-quality development zones, secondary development zones, balanced development zones, and development potential zones; see Figure 11. For high-quality development zones, the specific requirements are that the interaction level of the triangle model is high (Area C), the coupling degree is at the high-level coupling stage (Stage 4), and the coupling coordination degree is at the intermediate coordination stage (Stage 8); this classification included Wuhan. Secondary development areas exhibit high production and living functions, with a high coupling coordination and coupling degree suitable for living and production, and they have a certain radiation effect on the surrounding areas. The specific requirements are that the interaction level of the triangle model is at a low (D area), the coupling degree is at the high-level coupling stage (Stage 4), and the coupling coordination degree is at the intermediate coordination stage (Stage 8). The divisions included Yichang, Xiangyang, and Jingmen. Balanced development areas represent regions with balanced functions and a high degree of coordination. The specific requirements are that the triangle model interaction level is low (Area D), the coupling degree is at the high-level coupling stage (Stage 4), and the coupling coordination degree is at the primary coordination stage (Stage6). The classified regions included Xiaogan, Jingzhou, Suizhou, Xianning, Xiantao, Qianjiang, and Huangshi. Development potential areas are regions with a low functional index, low production and living functions, and relatively low coordination. The specific requirements are that the triangle model interaction level is at a low (E area), the coupling degree is at the grinding stage (Stage 3) or above, and the coupling coordination degree is at the barely coordinated stage (Stage 7). The classification results included Enshi, Shiyan, Huanggang, and Tianmen, where in Enshi, the coupling degree is in the grinding stage and has relatively high development potential.

## 5. Discussion

### 5.1. Shortcomings and Prospects

The current research on the production-living-ecology functions suggests that all rely on the national land space or multifunctional land [67]. In addition, research on multifunctional land evaluation systems has become increasingly mature [68,69]. At the same time, with its development, in order to achieve intensive and efficient production spaces, livable and moderate living spaces, and beautiful ecological spaces, China has become a leader in evaluation systems for multifunctional land [70] and has gradually deepened its research on the production-living-ecology functions based on land space [71,72]. At present, research has focused on the definition of the concept of the production-living-ecology functions [73], the construction of indicator systems [74], the study of distribution characteristics [75], the study of influencing factors [76], and spatial and temporal characteristics [77]. However, not much attention has been paid to the two decades since the beginning of the 21st century, during which the country has undergone rapid development and intense conflict. In addition, less attention has been paid to large-scale research, for example, using provincial regions, instead focusing more on comparative analyses in time sequence. Few studies have been conducted on spatial characteristics, the construction of indicator systems is not uniform, and the generation of policy instruments after the corresponding assessment of the production-living-ecology functions is lacking. The main methods currently used to study the production-living-ecology functions include the entropy method, the coupled coordination evaluation model, and the spatial correlation analysis method, among others, which may be combined with more methods, such as machine learning [78] and artificial intelligence [79]. The data source of the three-life function is also relatively singular, essentially based on statistical data combined with different scales of remote sensing [80,81]. The data obtained from different scales of remote sensing may be able to enrich the research content. Most of the existing methods focus on the study of the characteristics of the production-living-ecology functions themselves, and there is less research on the coupling and coordination characteristics and interrelationships among them. In addition, in the entropy method, the production-living-ecology functions are essentially used separately, as this method defaults to the production-living-ecology functions having the same weights, ignoring the objectivity of the interrelationships among them. Therefore, this study adopted the global entropy value method to objectively evaluate the weight of the production-living-ecology functions, obtain information from the data, determine the interrelationships among the functions, and conduct corresponding investigations, including coupling and coordination evaluation, on this basis. The innovation of this paper mainly lies in the fact that the global entropy value method explains more comprehensively and objectively than the general entropy value method the changes of various production-, life-, and ecology-related indicators under the index of the production-living-ecology functions in Hubei Province, in order to obtain an objective quantitative assessment of the development status of the indicators related to the production-living-ecology functions in Hubei Province, and thus to conduct a dynamic study of the comprehensive triangle model and the coupled coordination degree model of the production-living-ecology functions in Hubei Province. In addition, at the level of the entropy method, analyses of the last 20 years of the production-living-ecology functions for as many as 16 regions under the provincial administrative regions from 2000 to 2020 provide a large amount of new information, which is more meaningful for the current five-year period of planning.

In this study, we investigated the degree of coupling and coordination of the production-living-ecology functions in Hubei Province from 2000 to 2020 and constructed an index system. The results of the study showed that since the beginning of the 21st century, Hubei Province has entered a high-speed development stage, characterized by an increasingly rapid development rate from 2000 to 2015 and a slower growth rate in 2020. In addition, the production-living-ecology functions gradually entered a coordinated development stage.

According to the production-living-ecology function development status of different areas in the province, zoning management divisions were laid out and targeted to achieve overall coordinated development and promote the sustainable use of national spatial resources. This study has the following shortcomings: the data were not easy to obtain, only a five-year period was selected, and the years were not continuous. The data used in subsequent studies should ideally be more accurate, the weights of the objective differences in the index system should be calculated in a normalized way, and a better optimization calculation method should be utilized. In addition, in this study, only Hubei Province was selected as the research region; we hope that future studies can be conducted at the macroscopic national scale, the world scale, and the microscopic town and village scale. Finally, the production-living-ecology function theories have not been studied deeply enough; we plan to construct a recognized theory and index system in follow-up research.

### 5.2. Policy Recommendations

For the high-quality development area, measures should be taken to maintain its development advantages and enhance its ecological functions, with the Wuhan area in eastern Hubei Province focusing on optical core screen end networks, biomedicine, and other fields to lead the high-quality development of emerging industries, continue to become the most important growth pole of cities in the middle reaches of the Yangtze River, and drive the development of other surrounding areas, such as Xiaogan, Ezhou, Huanggang, Xianning, Xiantao, Tianmen, and Qianjiang. Wuhan needs to continue to develop the modern service industry based on the “four banks of the two rivers” concept; concentrate on the development of the national financial center; and take advantage of local industry, including the national leading transportation advantage, the export of talents from the university city, the centralized distribution of various industrial parks, etc., in order to develop new industrial centers and build intelligent industrial clusters, such as the automobile industry, electronic and electrical industry, etc. At the same time, the construction of the Yangtze River ecological corridor, the Han River ecological corridor, many ecological wetlands such as East Lake and South Lake, and ecological green areas, such as Mulan Grassland, guarantee the steady development of ecological functions.

For the secondary development areas, measures should be taken to optimize development and give full play to regional advantages. For the Xiangyang area, these measures should focus on new energy vehicles and equipment manufacturing. The northern core of western Hubei province, to undertake the north–south corridor of western Hubei province, can take advantage of railroad and highway transportation hubs to drive the development of Shiyan, Suizhou, etc., while building ecological barriers, such as ecological protection forests to protect the ecological function area of Qinba biodiversity Ecological balance. For Yichang and Jingmen in the northern core of Hubei province, these measures should focus on bio-agriculture and advanced chemical materials. The southern core of the western part of Hubei Province, to undertake the east–west channel in the western part of Hubei Province, can use the convenience of the Yangtze River waterway and the Han River waterway for traffic to drive the development of Jingzhou, Tianmen, Qianjiang, Xiantao, Enshi, and other areas. At the same time, the southern core and the northern core will achieve synergistic development.

For balanced development areas, such as Xiaogan, Ezhou, Jingzhou, Suizhou, Xianning, Xiantao, Qianjiang, and Huangshi, measures should be adopted to find regional advantages and characteristics, promote industrial radiation from high-quality living areas and secondary development areas, build provincial strategic emerging industry clusters with distinctive characteristics and staggered development, become the backbone of the province’s development, and enhance the production-living-ecology functions in all aspects. Qianjiang, for example, combined with two high-speed systems (Hanyi high-speed and Qian Shi, Qian Zao high-speed), two railways (Hanyi passenger high-speed railroad and Qianjiang freight rail spur), two waterways (Han River and Jianghan Canal), and two ports (Qianjiang Port Zekou port area and Hongqi port area) as the support of the public, rail, and water intermodal modern integrated transportation system, combined with the local characteristics of the water garden, and the advantages of the crayfish industry, to create the crayfish with rice industry as the representative of the industry Cluster, the use of its own oilfield resources, to create local characteristics of new energy, new chemicals, new materials industry clusters.

For development potential, areas, such as Enshi, Shiyan, Huanggang, and Tianmen, while giving play to their own ecological characteristics, such measures should create cultural and tourism industries, strengthen the construction of transportation, and promote the radiation of living and production functions from surrounding areas to improve the living level and production level, create livable ecological cities, attract people from other areas for recreation and tourism, and achieve overall regional balance. Enshi, for example, can take advantage of the local situation and the junction of Hubei Province, Hunan Province, and Chongqing City to develop transportation, become a major transportation route of three provinces, combine local selenium-rich characteristics, build the world capital of selenium industry, vigorously develop selenium-rich tea and selenium-rich recreation tourism, etc.

The overall approach should be multi-core-driven and multi-influential, forming an integrated and interactive development system in Hubei Province, ultimately realizing the full development and coordination of the production-living-ecology functions. The development management zoning is conducive to the realization of rural revitalization, the reduction of the differences among the geographical areas of Hubei Province, and the realization of common prosperity under the coordination of the production-living-ecology functions.

## 6. Conclusions

Taking Hubei Province of China as an example, an indicator system was constructed and the global entropy value method, triangle model, and coupled coordination degree model were applied to analyze the degree of conflict and coordination among its production-living-ecology functions from 2000 to 2020. The results showed a dynamic change, and the four main conclusions are as follows:

(1) With respect to the time scale, along with the shift of China’s development center, Hubei Province (located in the central region of China) has undergone considerable development. The indices of the production-living-ecology functions have improved year by year, and the degree of coordination of the production-living-ecology functions has increased year by year, from the stage of dysfunction with a certain degree of conflict to the stage of coordination with a high level of coupling.
(2) At the spatial scale, the development of the production-living-ecology functions in Hubei Province has been unbalanced, which is likely related to the overall development strategy of “two circles and one belt” in Hubei Province. The eastern part of Hubei Province has a higher degree of coordination of the production-living-ecology functions, while the western part has a lower degree of coordination of the production-living-ecology functions. Centering on the Yangtze River Economic Belt in Hubei Province, Wuhan City Circle with Wuhan City as the center and the West Hubei Ecological and Cultural Tourism Circle have been formed
(3) Among the production-living-ecology functions, the ecological function of Hubei Province as a whole has undergone minimal changes and maintained stable development, while the living and production functions have developed greatly, indicating that the balance of the ecological function has not been destroyed by the development of the living and production functions in Hubei Province during the period from 2000 to 2020 and the orderly development of the ecological environment has been protected in the process of urbanization and new industrialization.
(4) According to the development and coordination of the production-living-ecology functions of each region in Hubei Province, they were divided into high-quality development areas, secondary development areas, balanced development areas, and development potential areas, and development suggestions were provided to give full play to the advantages and make up for the shortcomings of these regions according to their own characteristics to achieve a balance of the production-living-ecology functions across the province.

## Figures and Tables

**Figure 9:**
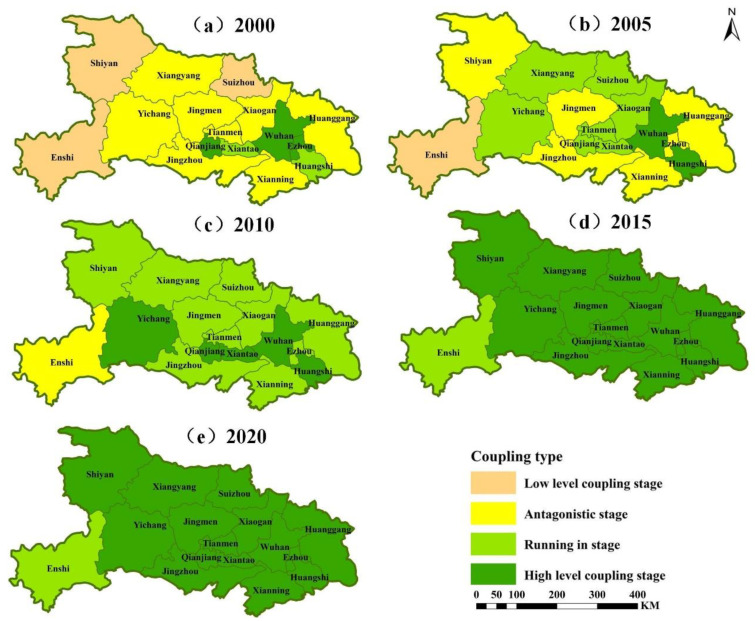
Change in the coupling degree of the production-living-ecology functions in Hubei Province from 2000 to 2020.

**Table 1:** Table of the production-living-ecology function indicators and their corresponding weights.

First Level Indicator	Weights of Primary Indicators	Secondary Indicator	Secondary Index Weight	Third Level Indicator	Third Level Indicator Weight
Production function index (PFI, C1)	0.3229	Agricultural production function (F1)	0.0946	Per capita gross agricultural product (yuan)	0.0352
				Per capita gross fishery production value (yuan)	0.0594
		Economic growth function (F2)	0.1396	Per capita GDP (yuan)	0.0603
				Per capita financial income of cities and prefectures (yuan)	0.0793
		Traffic function (F3)	0.0887	Average ground highway passenger volume (person/km^2^)	0.0349
				Total average ground road freight volume (t/km^2^)	0.0538
Living function index (LFI, C2)	0.6403	Employment security function (F4)	0.1058	Number of employees per unit (person/km^2^)	0.0583
				Per capita annual income of farmers (yuan)	0.0475
		Social security function (F5)	0.123	Per capita investment in fixed assets of the whole society (yuan)	0.0647
				Per capita municipal and state financial expenditure (yuan)	0.0583
		Habitat function (F6)	0.1752	Per capita real estate development investment (yuan)	0.1047
				Proportion of built-up area in administrative regions	0.0705
		Entertainment function (F7)	0.0995	Public book collection per capita (volume, piece)	0.0508
				Total retail sales of consumer goods per capita (yuan)	0.0487
		Science and education function (F8)	0.0536	Proportion of education expenditure in public budget (%)	0.0102
				Proportion of scientific expenditure in public budget (%)	0.0434
		Medical security function (F9)	0.0832	Number of hospitals per 10,000 people	0.0547
				Number of hospital beds per capita	0.0285
Ecology function index (EFI, C3)	0.0369	Resource security function (F10)	0.0133	Greening coverage rate of built-up area	0.0047
				Population density (person/km^2^)	0.0086
		Ecological balance function (F11)	0.0236	Per capita sown area of crops (m^2^)	0.0104
				Per capita industrial sulfur dioxide emissions (kg)	0.0056
				Fertilizer application amount per hectare (net amount, ton)	0.0045
				Carbon emissions per capita (kg)	0.0031

**Table 2:** Interaction horizontal partition of the triangle model.

Category	Index Range			Multifunctional Level
	NLFI	NNPFI	NNEFI	
A	0.8–1.0	0–0.2	0–0.2	Very high
B	0.6–0.8	0–0.4	0–0.4	High
C	0.4–0.6	0–0.6	0–0.6	Higher
D	0.2–0.4	0–0.8	0–0.8	Lower
E	0–0.2	0–1.0	0–1.0	Low

Abbreviations: LFI—normalized living function index; NPFI—normalized non-production function index; NEFI—normalized non-ecology function index; NEFI—normalized non-ecology function index.

**Table 3:** Table of the evolutionary direction of the production-living-ecology functions.

Direction Change Range (°)		Index Development Direction			Multifunctional Evolution Trend
		NLFI	NNPFI	NNEFI	
T1	N0–60	↑	↑	↓	General upward trend
T2	N60–120	↑	↓	↓	Strong upward trend
T3	N120–180	↑	↓	↑	General upward trend
T4	N180–240	↓	↓	↑	General downward trend
T5	N240–300	↓	↑	↑	Strong downward trend
T6	N300–360	↓	↑	↓	General downward trend
T7	Unchanged	-	-	-	General downward trend

Abbreviations: LFI—normalized living function index; NPFI—normalized non-production function index; NEFI—normalized non-ecology function index; NEFI—normalized non-ecology function index.

**Table 4:** Coupling degree type division.

Coupling Range	Coupling Type
(0, 0.3]	Low-level coupling stage
(0.3, 0.5]	Antagonistic stage
(0.5, 0.8]	Running-in stage
(0.8, 1]	High-level coupling stage

**Table 5:** Classification standard of coupling coordination degree.

D Value Range	Coordination Level	Coupling Coordination Degree
(0.0~0.1)	1	Extreme maladjustment
[0.1~0.2)	2	Severe maladjustment
[0.2~0.3)	3	Moderate maladjustment
[0.3~0.4)	4	Mild maladjustment
[0.4~0.5)	5	On the verge of maladjustment
[0.5~0.6)	6	Grudging coordination
[0.6~0.7)	7	Primary coordination
[0.7~0.8)	8	Intermediate coordination
[0.8~0.9)	9	Good coordination
[0.9~1.0)	10	High-quality coordination

**Table 6:** Table of coupling and coordination.

Area	Coupling Degree and Coupling Coordination Value	Year				
		2000	2005	2010	2015	2020
Hubei	C	0.44	0.574	0.798	0.954	0.952
	D	0.342	0.384	0.506	0.652	0.694
Wuhan	C	0.989	0.997	0.925	0.87	0.841
	D	0.455	0.497	0.625	0.793	0.779
Huangshi	C	0.769	0.886	0.952	0.996	0.992
	D	0.425	0.48	0.497	0.591	0.628
Shiyan	C	0.26	0.415	0.596	0.826	0.843
	D	0.283	0.339	0.446	0.583	0.624
Yichang	C	0.446	0.666	0.834	0.976	0.958
	D	0.365	0.414	0.531	0.718	0.748
Xiangyang	C	0.485	0.519	0.776	0.961	0.914
	D	0.373	0.364	0.49	0.673	0.704
Ezhou	C	0.882	0.45	0.555	0.832	0.959
	D	0.487	0.29	0.341	0.595	0.68
Jingmen	C	0.345	0.463	0.715	0.894	0.887
	D	0.334	0.375	0.515	0.649	0.713
Xiaogan	C	0.409	0.571	0.77	0.963	0.937
	D	0.335	0.365	0.462	0.567	0.612
Jingzhou	C	0.389	0.437	0.697	0.875	0.887
	D	0.324	0.32	0.473	0.594	0.638
Huanggang	C	0.317	0.406	0.714	0.863	0.879
	D	0.264	0.294	0.417	0.544	0.576
Xianning	C	0.308	0.458	0.695	0.877	0.885
	D	0.299	0.353	0.484	0.62	0.648
Suizhou	C	0.293	0.541	0.759	0.837	0.881
	D	0.279	0.366	0.474	0.56	0.593
Enshi	C	0.184	0.207	0.392	0.643	0.704
	D	0.226	0.247	0.36	0.495	0.553
Xiantao	C	0.578	0.678	0.808	0.92	0.947
	D	0.358	0.438	0.512	0.622	0.648
Qianjiang	C	0.814	0.714	0.858	0.958	0.964
	D	0.309	0.423	0.526	0.634	0.69
Tianmen	C	0.391	0.541	0.725	0.884	0.894
	D	0.357	0.362	0.458	0.575	0.59

**Table 7:** Classification standard of coupling coordination degree.

Partition Type	Horizontal Zoning of Triangle Model	Coupling Degree Type	Coupling Coordination Degree
High-quality development zone	C	4	8
Secondary development zone	D	4	8
Balanced development zone	D	4	7
Development potential zone	E	≥3	6

## Data Availability

Not applicable.
